# Mineral sources in natural habitats for a leaf-eating primate

**DOI:** 10.1093/cz/zoag004

**Published:** 2026-01-29

**Authors:** John F Aristizabal, Nicoletta Righini, Sergio Albino-Miranda, Karla L Tapia-Fierro, Cuauhcihuatl Vital-García, Jessica M Rothman, Juan Carlos Serio-Silva

**Affiliations:** Departamento de Ciencias Químico-Biológicas, Instituto de Ciencias Biomédicas, Universidad Autónoma de Ciudad Juárez, Anillo Envolvente del PRONAF y Estocolmo s/n., Ciudad Juárez 32310, Chihuahua, Mexico; Departamento de Ciencias Veterinarias, Universidad Autónoma de Ciudad Juárez, Ciudad Juárez 32310, Chihuahua, Mexico; Instituto de Investigaciones en Comportamiento Alimentario y Nutrición (IICAN), Universidad de Guadalajara, Ciudad Guzmán 49000, Jalisco, Mexico; Red de Biología y Conservación de Vertebrados, Instituto de Ecología, A.C. (INECOL), Carretera antigua a Coatepec 351, Xalapa 91073, Veracruz, Mexico; Red de Biología y Conservación de Vertebrados, Instituto de Ecología, A.C. (INECOL), Carretera antigua a Coatepec 351, Xalapa 91073, Veracruz, Mexico; Departamento de Ciencias Veterinarias, Universidad Autónoma de Ciudad Juárez, Ciudad Juárez 32310, Chihuahua, Mexico; Department of Anthropology, CUNY Hunter College, 695 Park Ave, New York 10065, NY, USA; Red de Biología y Conservación de Vertebrados, Instituto de Ecología, A.C. (INECOL), Carretera antigua a Coatepec 351, Xalapa 91073, Veracruz, Mexico

**Keywords:** mineral intake, nutritional ecology, nutritional geometry, terrestrial feeding

## Abstract

Minerals are essential components of animal nutrition, playing a crucial role in health, reproduction, and survival. Studies show that mineral intake varies by species, habitat, and season. This study evaluates the mineral composition of food consumed by black howler monkeys (*Alouatta pigra*), an endangered primate in southeastern Mexico, considering differences among habitats (fragmented vs. conserved), strata (terrestrial vs. arboreal), seasons (rainy, dry, and *Nortes*), and food types. Leaves, fruits, and lianas (*N* = 84) were analyzed to estimate intake of key minerals (Ca, P, Mg, and K) in absolute (g/day) and proportional (% dry matter) terms. Results showed that foods from the forest fragment had higher concentrations of Ca (1.80 ± 1.06%) and Mg (0.37 ± 0.11%) and lower Na (0.01 ± 0.01) compared to the conserved forest (Ca: 0.76 ± 0.67%; Mg: 0.28 ± 0.1%; Na: 0.04 ± 0.01). Based on daily intake, Ca, P, and Mg were the most important. Seasonality influenced diet: during the *Nortes* season, Ca (2.20 g/day) and P (23.09 g/day) intake increased, linked to greater consumption of young leaves, with potential physiological implications due to their high concentrations. Mature leaves contributed over 50% of essential minerals like Ca, Mg, and Mn, while immature fruits were key sources of P and Cu. Ca intake in fragmented habitats was notably high, possibly with physiological implications. These findings underscore the role of dietary diversity in meeting mineral needs in fragmented environments. Despite mineral variation among foods, black howler monkeys attempted to meet requirements through varied food sources, reinforcing the importance of conserving plant diversity in fragmented ecosystems.

## Graphical Abstract

**Graphical Abstract:**
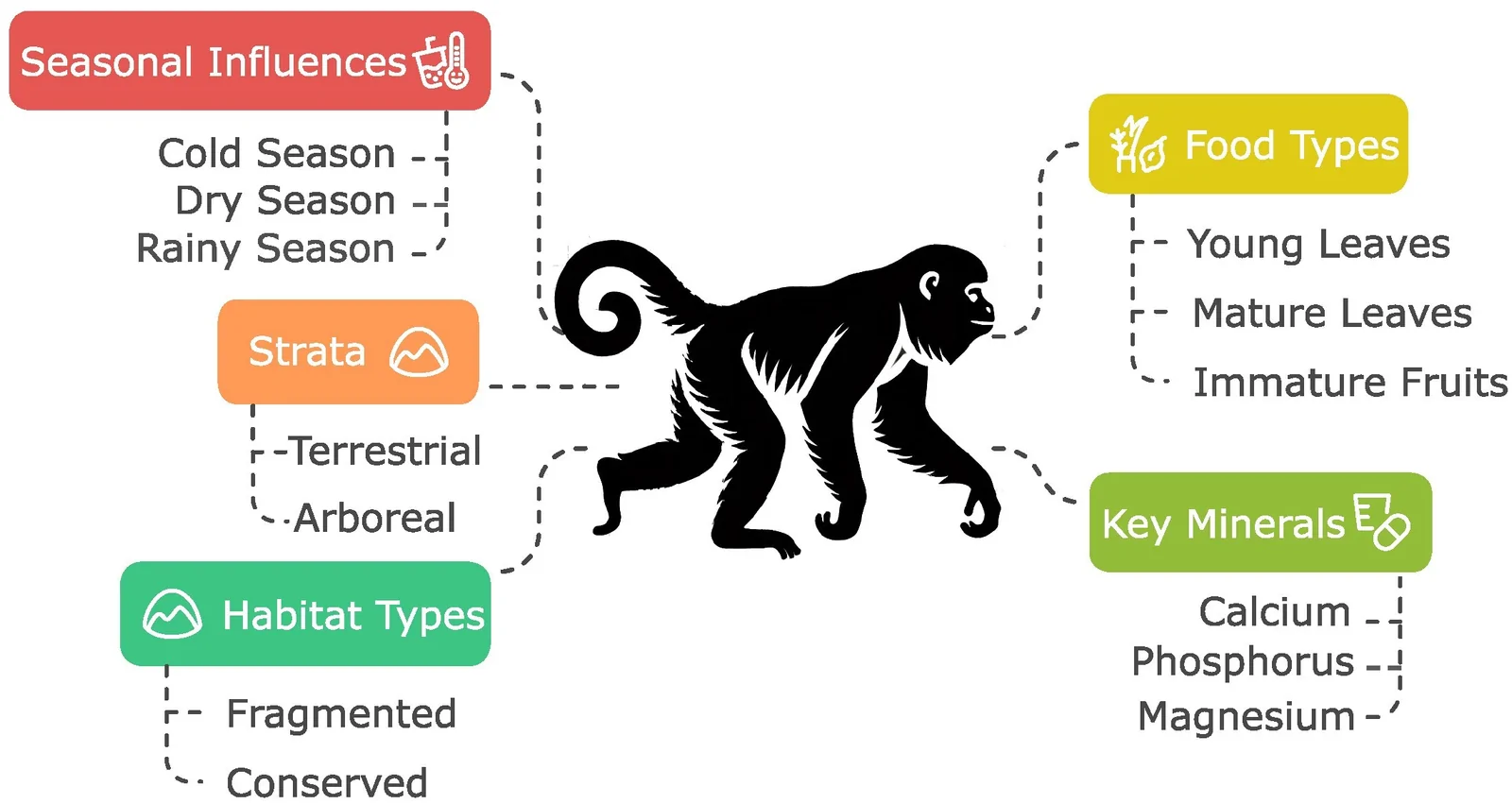

Minerals play a crucial role in animal nutrition and health, influencing a variety of physiological and behavioral functions (Barboza et al. 2009). Numerous studies have highlighted the importance of specific minerals for nonhuman primates, particularly calcium (Ca), sodium (Na), magnesium (Mg), iron (Fe), and phosphorus (P) (Cancelliere et al. 2014; Irwin et al. 2017; Grueter et al. 2018; Dunham and Opere 2021). Although required in relatively small amounts, these nutrients are essential for bone health maintenance, neuromuscular function, and hemoglobin synthesis, among other functions. In particular, macrominerals [such as Na, potassium (K), Ca, and P] are required by all vertebrates given their role as structural components of bone and teeth and their involvement in osmotic balance; whereas trace minerals, such as Fe, manganese (Mn), copper (Cu), and zinc (Zn) participate in biochemical pathways and have catalytic functions in activating enzymes and hormones (National Research Council 2003; Barboza et al. 2009). Consequently, maintaining an adequate mineral balance is critical in primate diets (Milton 2003), as habitat structure and its level of disturbance, seasonality, and sex-specific metabolic needs may affect mineral availability and intake. This underscores the need for a detailed understanding of mineral sources and intake in different ecological contexts (Irwin et al. 2017).

The mineral content of a food source can vary significantly depending on the plant species and the specific part consumed (McDowell 2003). Leaves, fruits, flowers, and bark exhibit variable mineral concentrations, requiring primates to carefully select the food they consume to meet their nutritional needs (Dunham and Opere 2021). In general, leaves contain higher concentrations of most minerals than fruits or flowers (Irwin et al. 2017). Additionally, mineral concentration depends on both the plant's geographic location and position in the canopy, with differences among trees, lianas, herbs, and epiphytes (Cancelliere et al. 2014; Irwin et al. 2017), adding another layer of complexity to primate feeding ecology. Seasonal variation in plant mineral composition can, in turn, translate into seasonal fluctuations in mineral intake, as primates modify their feeding behavior in response to changes in food availability (Rothman et al. 2008; Cancelliere et al. 2014). During periods of high fruit availability, primates have access to different plant nutritional profiles compared to drier seasons or times of lower fruit availability (Behie and Pavelka 2012). The variation in fruit consumption can lead to fluctuations in mineral intake, potentially impacting health and behavior (Rothman et al. 2008). Primates generally adjust their diet in response to seasonal food availability (Irwin et al. 2014), leading to significant differences in the intake of essential nutrients, such as Ca and Mg, across seasons (Cancelliere et al. 2014). Hence, primates may increase leaf consumption even during periods of high fruit availability, not as a substitute for fruits but as a complementary strategy to meet mineral requirements, given the higher mineral content of leaves relative to fruits (Raubenheimer et al. 2015). However, the expression of these dietary strategies ultimately depends on habitat structure and landscape context.

The fragmentation of tropical forests has increased significantly in recent decades as a result of deforestation driven by the conversion of natural areas into agricultural lands and cattle pastures (Pendrill et al. 2022). This process has disrupted both the continuity and quality of these ecosystems, with significant implications for biodiversity and the ecosystem services they provide (Newbold et al. 2015). Landscapes are increasingly composed of small forest remnants that harbor lower plant diversity compared to the original forest, including the loss of large trees, which are characterized by high primary productivity (Arroyo-Rodríguez and Mandujano 2006; Arroyo-Rodríguez and Dias 2010). However, the abundance and diversity of lianas in these habitats are high due to their rapid growth rate (Schnitzer and Bongers 2002). Several liana species have been identified as fallback foods in primate diets, defined as abundant, low-quality food resources consumed during periods of primary food scarcity (Marshall et al. 2009; Dunn et al. 2012). Although widely used, the concept of fallback foods remains debated in primate ecology, as such resources may also contribute substantially to nutritional intake beyond periods of primary resource scarcity (Lambert and Rothman 2015). Additionally, the consumption of unusual plant parts (i.e., those infrequently consumed, relative to the species’ typical diet under less disturbed conditions) may serve as a strategy to cope with limited availability of preferred resources and to compensate for nutritional deficiencies (Chaves and Bicca-Marques 2012). For example, studies on mantled howler monkeys (*Alouatta palliata*) in forest fragments in southeastern Mexico showed a high reliance on unusual food sources, such as lianas, shrubs, and herbs, which together accounted for one-third of total feeding time (Asensio et al. 2007).

Highly folivorous arboreal primates can exhibit terrestrial behaviors and use the landscape matrix to reach different forest fragments to supplement their diets (Bicca-Marques and Calegaro-Marques 1994; Harris and Chapman 2007; Pozo-Montuy et al. 2013). The reduction of the canopy cover in degraded habitats and the increase in ambient temperature promote greater terrestrial activity, either to move between forest patches, forage (on the ground and in lower strata, <1 m), or to regulate body temperature in warm climates (Eppley et al. 2016, 2022). However, this behavior could lead to an energy allocation conflict, as descending to the ground or foraging from shrubs can increase predation risk and energy expenditure, which must be justified by the rewards obtained (Pozo-Montuy et al. 2013). Therefore, although foods from this stratum may provide important nutritional benefits in fragmented environments, the mineral composition of non-arboreal food resources remains poorly understood in primate nutritional ecology.

The relationship between mineral consumption and the nutritional needs of primates has been studied in relatively few species, such as black howler monkeys (*A. pigra*: Behie and Pavelka 2012), diademed sifakas (*Propithecus diadema*: Irwin et al. 2017), colobus monkeys (*Colobus angolensis palliatus*: Dunham and Opere 2021), and, more recently, in Japanese macaques (*Macaca fuscata*: Hanya et al. 2023). These studies suggest that primates often face challenges in obtaining adequate levels of minerals, as many wild food sources fall below the recommended levels established for primates in captivity (Irwin et al. 2017; Dunham and Opere 2021). Such recommendations are based on physiological requirements derived from controlled feeding studies in managed primate colonies and summarized in the Nutrient Requirements of Nonhuman Primates (NRC 2003). In wild settings, primates may therefore engage in a variety of behavioral strategies to cope with mineral limitations, particularly for minerals such as Na, sometimes involving behavioral trade-offs or increased exposure to risk (Oates 1978; Magliocca and Gautier-Hion 2002; Fedurek et al. 2024). Consistent with this pattern, primates may forage selectively based on mineral content, favoring foods rich in essential elements such as Ca, P, and Na when available (Rothman et al. 2006; Behie and Pavelka 2012; Dunham and Opere 2021; Fedurek et al. 2024). However, environmental factors can significantly influence mineral availability and, consequently, primate dietary choices (Behie and Pavelka 2012). Nevertheless, mineral intake does not directly translate into absorption, as bioavailability and dietary interactions may modulate mineral utilization; for instance, iron absorption depends on its chemical form and on interactions with compounds such as tannins or fiber (Lambert and Rothman 2015). Further research is needed to better understand mineral requirements and their effects on wild primate populations.

Within this broader ecological and comparative context, black howler monkeys (such as *A. pigra*) are large-bodied atelids traditionally described as arboreal frugivore-folivores, with diets dominated by leaves and fruits and supplemented by flowers and other plant parts depending on seasonal availability (Dias and Rangel-Negrín 2015; Gisbrecht et al. 2025, 2026). Their feeding ecology has been described as part of an integrated dietary syndrome characteristic of the genus *Alouatta* (Garber et al. 2015). This syndrome combines a strong reliance on leaves with high dietary flexibility and energy-conserving behavioral and digestive adaptations that allow howlers to exploit fibrous and nutritionally variable foods. Although fruits may contribute substantially to energy intake when available, leaves remain a consistent dietary component and an important source of protein and minerals. These traits enable *A. pigra* to adjust food choice in response to seasonal variation and habitat disturbance, including forest fragmentation, without reliance on behaviors such as geophagy or mineral lick use.

Based on the above and considering their essential physiological functions and dietary relevance for primates, we examined the diet of six groups of black howler monkeys (*A. pigra*) in southeastern Mexico. First, we quantified the mineral content (Ca, P, Na, Fe, Zn, Mg, Mn, Mo, K, and Cu) of foods consumed by black howler monkeys (four groups in forest fragments <4 ha; two groups in a more conserved habitat of 2,100 ha) to determine variability according to food types, plant types, and seasons. Second, we assessed whether the concentrations of key minerals within part plants consumed by howler monkeys varied according to foraging stratum (arboreal vs. non-arboreal), and between foods from fragmented vs. conserved habitats. Third, we estimated mineral intake (in terms of percentage of dry matter [%DM] and absolute intake [g/day]) of three groups inhabiting forest fragments to identify the minerals that most contribute to daily intake in the diet of black howler monkeys, and to evaluate whether sex, group, and seasons influence this pattern.

## Materials and methods

### Study area

This study was conducted at two sites in southeastern Mexico, representing a fragmented habitat condition and a conserved habitat condition. The first site was located at the Primatological and Wildlife Research Station-PWRS in the municipality of Balancán, in the state of Tabasco, southeastern Mexico (17°46′52″N; 91°30′22.93″W). The site is part of the Usumacinta River wetland subsystem and is situated 30 m above sea level. The landscape consists of several forest fragments (<4 ha each, separated by distances ranging from 100 to 800 m) embedded within agricultural matrices dominated by pastures and livestock production. This landscape includes various types of vegetation, such as tropical dry forest, palm forest, riparian forest, and secondary forest with an average canopy height of 9.1 ± 3.9 m. The average monthly temperature ranges between 21.7 and 33.5 °C. The conserved site was located at El Tormento-ET (18°36′04.00″N; 90°48′03.00″W), 117 km away from PWR. ET is a 2,100-ha forested area owned by the Instituto Nacional de Investigaciones Forestales, Agrícolas y Pecuarias (INIFAP) in the state of Campeche. The dominant vegetation types in this area of the Yucatán Peninsula are seasonal dry forests and evergreen forests (Miranda and Hernández 1963), with an average canopy height of 18.4 ± 4.7 m with trees up to 30 m.

At both sites, three distinct seasons can be identified: a rainy season from June to September with an average monthly precipitation of around 300 mm and high availability of mature leaves and fruits; a *Nortes* season, characterized by strong cold winds from October to January with an average monthly precipitation between 100 and 200 mm, and high availability of young leaves and lower fruit availability; and a dry season from February to May, with an average monthly precipitation below 100 mm, and high availability of mature leaves (Aristizabal et al. 2019; CONAGUA, 2025).

### Behavioral data and diet composition

To analyze the feeding behavior of black howler monkeys, we studied six groups. In the fragmented habitat (PWRS), four groups were monitored over different years (one of the groups was followed during two stages), while in the conserved habitat (ET), two groups were studied. These observations were divided into three stages based on the characteristics of the study sites rather than chronological order. Stage 1: Two groups in PWRS were studied for a total of 658 observation hours. Each group inhabited a different forest fragment (2.7 ha and 3.9 ha, respectively). Data collection took place from August 2012 to March 2013 (see Aristizabal et al. 2017). Stage 2: Three groups in PWRS were monitored for 1,253 observation hours. Group 1 (same as in Stage 1) occupied a 2.7-ha fragment, while Group 2 and Group 3 inhabited fragments of 0.8 ha and 3.2 ha, respectively. Observations occurred from June 2016 to May 2017 (see Aristizabal et al. 2019). Stage 3: Two groups were observed for 1,300 h in a conserved habitat (a 2,100-ha forested area in ET) between September 2010 and November 2011 (see Righini et al. 2017).

We conducted continuous focal follows of a single individual throughout the day (≈12 h per session), with the following number of focal sessions: Stage 1 *n* = 46, Stage 2 *n* = 124, Stage 3 *n* = 91. We recorded the time spent feeding, resting, moving, and other activities (e.g., playing, vocalizing, and agonistic encounters). For this study, we defined non-arboreal stratum use (ground and lower strata, <1 m) following Eppley et al. (2022), where terrestriality refers to the extent to which an arboreal species utilizes the ground for various activities.

We recorded the food intake (gr) of five food categories that constituted more than 90% of the time spent feeding of howler monkeys’ diet (Aristizabal et al. 2017): mature leaves, young leaves, ripe fruits, unripe fruits, and flowers. We first estimated the feeding rate (units of food consumed per minute) for each food item during each feeding event (Rothman et al. 2012; Aristizabal et al. 2017). We then multiplied this rate by the total time spent feeding on each item to calculate the number of units consumed per day (see Aristizabal et al. 2017).

To calculate the percentage of dry weight intake (Dry matter-DM in grams), we collected samples directly from tree canopies using 15-m poles and weighed 100 units of each food item to estimate fresh mass consumption within a maximum of 5 days from the first observed consumption of a new food (Rothman et al. 2012). Additionally, we gathered 50 to 250 g of fresh material per food item, depending on the type and moisture content (e.g., flowers, leaves), and dried the samples in a cool, dark room at ambient temperature (ranging from 21.7 °C to 33.5 °C). To prevent fermentation and mold in fruits, we either cut them into pieces or used a food dehydrator (NESCO-FD-2000) at 55 °C until the sample reached a constant weight. This process allowed us to determine the moisture percentage of each food item. We then subtracted this value from the wet weight recorded during feeding events to estimate the dry weight intake (grams) (Rothman et al. 2012). To calculate daily mineral intake (g/day), we followed the approach of Irwin et al. (2017). We summed the DM intake of all food items across feeding bouts per day and multiplied it by the corresponding mineral concentration (percentage of DM) (see details in Irwin et al. 2017). The results are presented both proportionally (% DM) and in absolute terms (g/day).

### Mineral analysis

Food samples collected during the Stage 1 and 3 were analyzed for mineral compositions (Ca, P, Mg, K, Na, Fe, Zn, Cu, Mn, and Mo) at Dairy One Forage Lab (Ithaca, New York, USA). Samples were ashed in a muffle furnace at 500 °C for 4 hr, then ash residue was suspended in 3 mL of 6N HCl and evaporated to dryness on a 100–120 °C hot plate. Minerals were then extracted with an acid solution (1.5N HNO3 + 0.5N HCl) and analyzed using a Thermo Jarrell Ash IRIS Advantage HX Inductively Coupled Plasma (ICP) Radial Spectrometer. For the analysis of food samples from Stage 2, the plant material was dried in an oven at 60 °C for 24 h, and then all tissues were ground (particle size of 1.0 mm). Afterward, we used 0.5 g of plant tissue with an acid solution (2 HClO4 mL + 10 mL HNO3) and performed a microwave-assisted digestion, using a CEM microwave oven model MARS 6 under the following conditions: a temperature of 210 °C for 20 min, with a 30 min pause (Altundag et al. 2011). The concentration of minerals (Ca, Mg, Fe, Cu, Zn, and Mn) in foods from Stage 2 was quantified using a Varian Atomic Absorption Spectrophotometer (AAS) model 240FS equipped with an autosampler and deuterium lamps at the Instituto de Ecología, A.C. soil laboratory. These analyses provided mineral composition data used to evaluate variation across food types, strata, habitats, and seasons (Objectives 1 and 2).

### Data analysis

To characterize mineral composition in the foods consumed by black howler monkeys, we first quantified the concentration of each mineral across all food items collected during the study. Mean concentrations and variability (±SD) were summarized according to food type (flowers, immature fruits, mature fruits, young leaves, mature leaves, and petioles), plant type (lianas vs. trees), foraging stratum (arboreal vs. non-arboreal), habitat condition (fragmented vs. conserved), a season (rainy, dry, and *Nortes*). Differences in mineral concentrations among these categories were evaluated using Mann–Whitney *U* tests. To identify which minerals contributed most strongly to daily mineral intake, we conducted a principal component analysis (PCA; Greenacre et al. 2022) using intake data expressed as percentage of dry matter (% DM). This analysis was performed exclusively using data from groups inhabiting forest fragments (Stage 2), as this stage provided the complete intake dataset. The PCA was used to evaluate the relative contribution of each mineral to the diet and to identify those with the greatest contribution to overall intake. We conducted the PCA using the FactoMineR package and visualized the results with factoextra (Le et al. 2008) in R software version 3.2.0 (R Core Team 2024). Based on PCA loadings in the first two dimensions, Ca, Mg, and P were identified as the most influential minerals and were used to construct equilateral mixture triangles (see Raubenheimer 2011) to visualize the nutritional space of arboreal versus non-arboreal foods, and foods from fragmented versus conserved habitats.

Finally, we quantified daily mineral intake (g/day) for each individual living in forest fragments (Stage 2; *N* = 124 focal days) and evaluated the effects of sex, season, and group using generalized linear models (GLMs) fitted with gamma error distributions, which are well-suited for positively skewed continuous data with a constant coefficient of variation (Cayuela Delgado and De La Cruz Rot 2022). For the GLMs, the response variable was the daily intake (g/day) of each mineral, and the explanatory variables included sex, season, and group. A model was considered plausible if its Δi value (the difference between the Akaike Information Criterion [AIC] of a given model and the lowest AIC value) fell between 0 and 2 (Grueber et al. 2011). If multiple models met this criterion, we selected the one with the highest cumulative Akaike weight (Σwi), provided it exceeded 0.6 (Burnham and Anderson 2002). For the best-selected model, we estimated the slopes (β coefficients) and calculated the explanatory percentage between levels using the exponential of the coefficients. Since we used repeated measures from the same individuals, we initially considered generalized linear mixed models (GLMMs). However, we encountered a “singularity” warning, which suggests overfitting due to an insignificant random effect. In such cases, it is recommended to simplify the model by removing the random factor (Bolker et al. 2009). Pairwise comparisons between factor levels (e.g., seasons, sex) were extracted directly from each GLM, which provided estimated differences, standard errors, and *P*-values. No additional post hoc tests or *P*-value corrections were applied, as each model addressed a specific response variable and set of predictors. All analyses were conducted in R version 3.2.0 using the nlme and bbmle packages (Pinheiro et al. 2016; Bolker and R Core Team 2017; R Core Team 2024).

## Results

### Mineral composition of foods consumed by black howler monkeys

Mineral concentrations varied widely among foods consumed by black howler monkeys and were influenced by food type, plant phenophase, season, habitat (conserved vs. fragmented), and foraging stratum (arboreal vs. non-arboreal). Macrominerals (Ca, P, Mg, Na, and K) from 84 food samples are reported as percentage of dry matter (% DM), whereas trace minerals (e.g., Cu, Zn, Fe, Mn, Mo) are expressed in mg/kg DM, following standard conventions in nutritional ecology (Rothman et al. 2012; Irwin et al. 2017) (Table 1).

**Table 1:** Variation in mineral content of foods consumed by black howler monkeys inhabiting forest fragments in Mexico. Means ± standard deviations are shown. FL: Flowers, MF: Mature fruits, IF: Immature fruits, YL: Young leaves, ML: Mature leaves, Pet: Petioles.

Habitat/Mineral	All foods	Food type						Plant type		Season		
		FL	MF	IF	YL	ML	Pet	Liana	Tree	Rainy	Dry	Nortes
**Fragmented-stage1** **2012–13**												
Cu (mg/Kg)	10.95 ± 5.01	—	9 ± 1.41	12 ± 9.54	11.63 ± 5.68	9.83 ± 2.86	13	11.4 ± 5.15	10.5 ± 5.10	9.4 ± 3.66	11 ± 5.66	12.88 ± 6.2
Mn (mg/Kg)	37 ± 38.18	—	13.5 ± 3.54	6.33 ± 2.52	55 ± 49.43	40.5 ± 25.70	11	47.4 ± 47.02	26.6 ± 25.02	26.1 ± 16.5	24.5 ± 29	53.8 ± 54.3
Fe (mg/Kg)	82.1 ± 84.97	—	27.5 ± 16.26	23 ± 16.46	136.6 ± 110	65.67 ± 37.9	31	121.3 ± 103	42.9 ± 34.67	67.3 ± 54.2	27 ± 19.80	114.4 ± 116
Zn (mg/Kg)	36.4 ± 31.21	—	17 ± 2.83	11 ± 3.61	59.4 ± 36.13	25 ± 18.15	36	57.5 ± 32.35	15.3 ± 4.57	36.1 ± 33.2	12 ± 0	42.9 ± 31.8
P (%)	0.27 ± 0.11	—	0.24 ± 0.13	0.16 ± 0.06	0.31 ± 0.10	0.29 ± 0.11	0.44	0.34 ± 0.10	0.20 ± 0.07	0.27 ± 0.12	0.17 ± 0.07	0.30 ± 0.11
Ca (%)	1.80 ± 1.06	—	1.25 ± 0.81	0.37 ± 0.34	2.35 ± 0.89	2.01 ± 1.04	1.51	2.34 ± 0.82	1.25 ± 1.02	1.65 ± 0.99	1.32 ± 1.56	2.11 ± 1.11
Mg (%)	0.30 ± 0.16	—	0.17 ± 0.09	0.11 ± 0.05	0.41 ± 0.08	0.28 ± 0.14	0.3	0.37 ± 0.11	0.23 ± 0.15	0.25 ± 0.12	0.22 ± 0.13	0.38 ± 0.15
Na (%)	0.01 ± 0.01	—	0.005 ± 0.002	0.008 ± 0.005	0.02 ± 0.01	0.01 ± 0.01	0.008	0.02 ± 0.01	0.01 ± 0.01	0.008 ± 0.006	0.022 ± 0.03	0.02 ± 0.01
K (%)	2.61 ± 1.601	—	1.72 ± 0.97	1.65 ± 0.49	3.57 ± 1.80	1.72 ± 0.76	5	3.71 ± 1.60	1.52 ± 0.47	2.47 ± 1.52	1.33 ± 0.13	3.12 ± 1.81
Mo (Mg/kg)	0.76 ± 1.04	—	1 ± 0	0.1 ± 0.09	1.24 ± 1.39	0.58 ± 0.42	0.5	1.19 ± 1.24	0.43 ± 0.35	1.06 ± 1.18	0.26 ± 0.35	0.64 ± 0.73
**No. samples**	**20**	—	**2**	**3**	**8**	**6**	**1**	**10**	**10**	**10**	**8**	**2**
**Fragmented-stage2** **2016–17**												
Cu (mg/Kg)	8.7 ± 6.04	5.81 ± 0.9	10.11 ± 6.67	11.37 ± 6.83	9.14 ± 4.12	6.35 ± 4.31	29.2	10.09 ± 9.50	8.43 ± 5.303	9.28 ± 6.56	8.17 ± 6.38	8.66 ± 5.38
Mn (mg/Kg)	29.84 ± 34.6	26.22 ± 6	12.54 ± 9.61	10.76 ± 7.19	32.16 ± 19.9	44.09 ± 48.	10.23	32.85 ± 25.6	29.27 ± 36.3	25.16 ± 17.4	43.11 ± 51.95	18.91 ± 13
Fe (mg/Kg)	47.9 ± 31.34	59.04 ± 45	22.29 ± 14.53	28.59 ± 41.3	44.38 ± 18.4	60.54 ± 25.3	35.19	68.83 ± 35.9	43.71 ± 29.2	47.89 ± 34.66	51.5 ± 28.79	42.7 ± 32.4
Zn (mg/Kg)	22.23 ± 20.2	32.2 ± 4.3	11.42 ± 9.01	15.61 ± 7.75	26.1 ± 18.01	20.87 ± 24	78.87	42.56 ± 37.2	18.39 ± 12.7	26.24 ± 24.97	19.93 ± 17.44	20.4 ± 17.9
P (%)	0.27 ± 0.07	0.3 ± 0.09	0.222 ± 0.18	0.23 ± 0.10	0.318 ± 0.18	0.161 ± 0.05	0.46	0.22 ± 0.12	0.23 ± 0.13	0.22 ± 0.13	0.23 ± 0.12	0.24 ± 0.14
Ca (%)	1.64 ± 1.52	0.72 ± 1.2	0.8 ± 1.01	0.77 ± 1.0	0.92 ± 1.16	2.81 ± 1.39	1.05	2.6 ± 1.61	1.45 ± 1.46	2.01 ± 1.1	2.25 ± 1.8	0.45 ± 0.79
Mg (%)	0.4 ± 0.2	0.42 ± 0.2	0.32 ± 0.18	0.18 ± 0.27	0.32 ± 0.07	0.50 ± 0.42	0.42	0.63 ± 0.32	0.35 ± 0.13	0.44 ± 0.14	0.46 ± 0.25	0.26 ± 0.09
**No. samples**	**44**	**4**	**4**	**9**	**8**	**18**	**1**	**7**	**37**	**15**	**13**	**16**
**Conserved-stage3** **2010–11**												
Cu (mg/Kg)	9.86 ± 6.25	7	11 ± 6	12 ± 6.4	15 ± 7.94	5.71 ± 4.27	—	—	—	—	—	—
Mn (mg/Kg)	23.81 ± 23.4	105	8.30 ± 3.91	15.80 ± 8.23	17 ± 6.56	31.86 ± 17.3	—	—	—	—	—	—
Fe (mg/Kg)	47 ± 20.15	77	39.20 ± 16.32	43 ± 30.72	50.7 ± 11.6	49.57 ± 16.6	—	—	—	—	—	—
Zn (mg/Kg)	17.71 ± 9.98	30	10.80 ± 6.30	20.60 ± 7.37	25 ± 17.35	15.71 ± 8.12	—	—	—	—	—	—
P (%)	0.14 ± 0.08	0.29	0.13 ± 0.05	0.14 ± 0.09	0.23 ± 0.07	0.10 ± 0.06	—	—	—	—	—	—
Ca (%)	0.76 ± 0.67	1.58	0.27 ± 0.20	0.39 ± 0.27	0.41 ± 0.11	1.40 ± 0.69	—	—	—	—	—	—
Mg (%)	0.28 ± 0.12	0.46	0.18 ± 0.05	0.26 ± 0.14	0.27 ± 0.12	0.33 ± 0.10	—	—	—	—	—	—
Na (%)	0.04 ± 0.01	0.03	0.03 ± 0.01	0.04 ± 0.03	0.05 ± 0.03	0.05 ± 0.04						
K (%)	2.13 ± 0.57	2.65	2.01 ± 0.49	2.09 ± 0.31	2.65 ± 0.7	1.27 ± 0.59						
**No. samples**	**20**											

In bold the number of samples.

Differences were observed in the concentrations of key minerals such as Ca, Mg, and P among the different food types. Leaves were the main source of several minerals. Young and mature leaves emerged as the primary sources of Ca (mean 2.35 ± 0.89% and 2.81 ± 1.39%, respectively) and Fe (136.6 ± 110 mg/kg and 65.7 ± 37.9 mg/kg, respectively). In contrast, fruits showed comparatively higher concentrations of Mg (0.32 ± 0.18%) and P (0.50 ± 0.42%).

In forest fragments, lianas showed higher concentrations of Mn, Fe, Zn, Mg, Ca and K than tree-derived foods (Table 1). Seasonal differences were also observed. During the *Nortes* season, foods consumed in fragmented habitats exhibited higher P (0.30 ± 0.11%) and K (2.57 ± 1.81%) concentrations. In contrast, during the rainy season, foods contained lower Mg concentrations (0.25 ± 0.12%) compared to the *Nortes* season (0.38 ± 0.15%) (Table 1).

Clear differences emerged between habitat types. On average, foods from the fragmented habitat (Stages 1 and 2) showed higher absolute concentrations of Ca, Mg, P, K, and Fe than foods from the conserved habitat (Stage 3). For example, Ca concentrations were higher in the fragmented habitat (Stage 1: 1.80 ± 1.06%; Stage 2: 1.64 ± 1.52%) than in the conserved habitat (Stage 3: 0.76 ± 0.67%). Similarly, Mg levels were higher in fragmented sites, particularly in leaves (Stage 1: 0.3 ± 0.16%; Stage 2: 0.4 ± 0.2%), compared to the conserved habitat (Stage 3: 0.28 ± 0.12%). P content was also markedly higher in fragmented habitats (Stage 1: 0.27 ± 0.11%; Stage 2: 0.27 ± 0.07%) than in the conserved habitat (Stage 3: 0.14 ± 0.08%). Fe concentrations followed a similar pattern, with higher values in fragmented areas (Stage 1: 82.1 ± 84.97 mg/kg; Stage 2: 47.9 ± 31.34 mg/kg) compared to the conserved habitat (Stage 3: 47.2 ± 20.55 mg/kg), indicating that young leaves and lianas in fragmented environments were rich in Fe sources. In contrast to other minerals, Na content was lower in fragmented habitats (Stage 2: 0.01 ± 0.01%) but higher in the conserved habitat (Stage 3: 0.04 ± 0.01%). Overall, these findings indicate that fragmented habitats can provide foods with higher concentrations of essential minerals (Ca, Mg, P, K, and Fe) compared to conserved environments.

### Minerals contributing most to variation in daily intake

We first used a principal component analysis (PCA) on daily mineral intake data (%DM) from groups inhabiting forest fragments to explore patterns of covariation among minerals. Dimensions 1 and 2 explained 43% and 27.1% of the total variance in mineral intake, respectively. Ca (eigenvalue = 0.49 -Dim1) and Mg (eigenvalue = 0.47 -Dim1) showed the highest loadings on Dimension 1, while P (eigenvalue = 0.57 -Dim2) was most strongly associated with Dimension 2 (Fig. 1).

**Figure 1:**
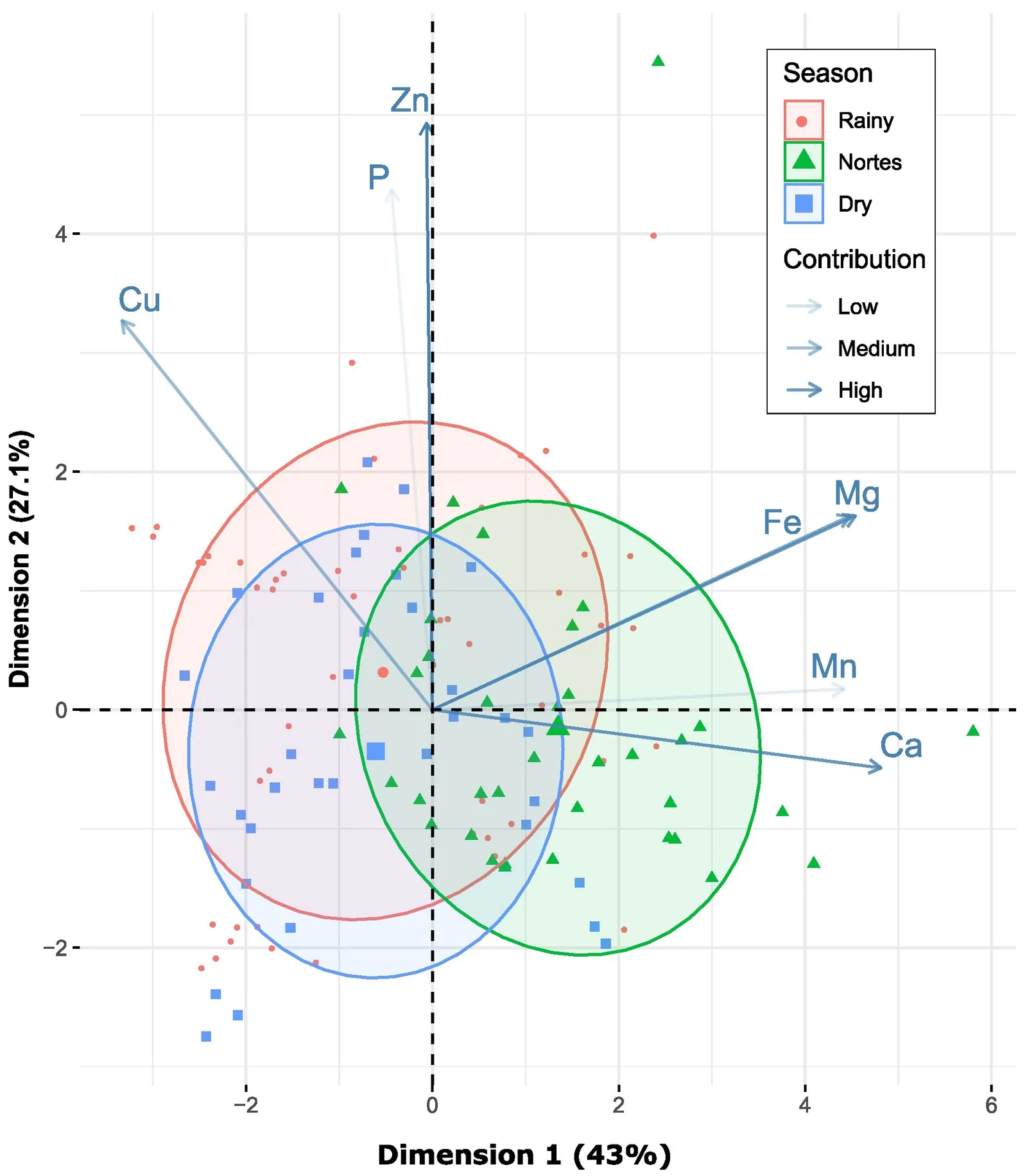
Biplot of PCA grouping the mineral intake (%DM) of black howler monkeys inhabiting forest fragments (stage 2). Each point represents 1 day of mineral intake. The contribution of the minerals (i.e., loadings in PCA) is represented by arrows.

Seasonal differences in mineral intake were reflected in the dispersion of observations in the PCA space, with days characterized by higher Ca and P intake clustering primarily during the *Nortes* season. Based on PCA loadings, Ca, Mg, and P were identified as the minerals contributing most strongly to variation in daily mineral intake and were therefore retained for subsequent graphical and statistical analyses.

### Nutritional space of foods by foraging stratum

Using Ca, Mg, and P (selected based on their contribution to variation in daily intake as identified by the PCA), we constructed equilateral mixture triangles to compare the relative mineral proportions of foods obtained from arboreal and non-arboreal strata. The nutritional spaces corresponding to foods from both strata largely overlapped, although the area representing arboreal foods was slightly larger (Fig. 2A).

**Figure 2:**
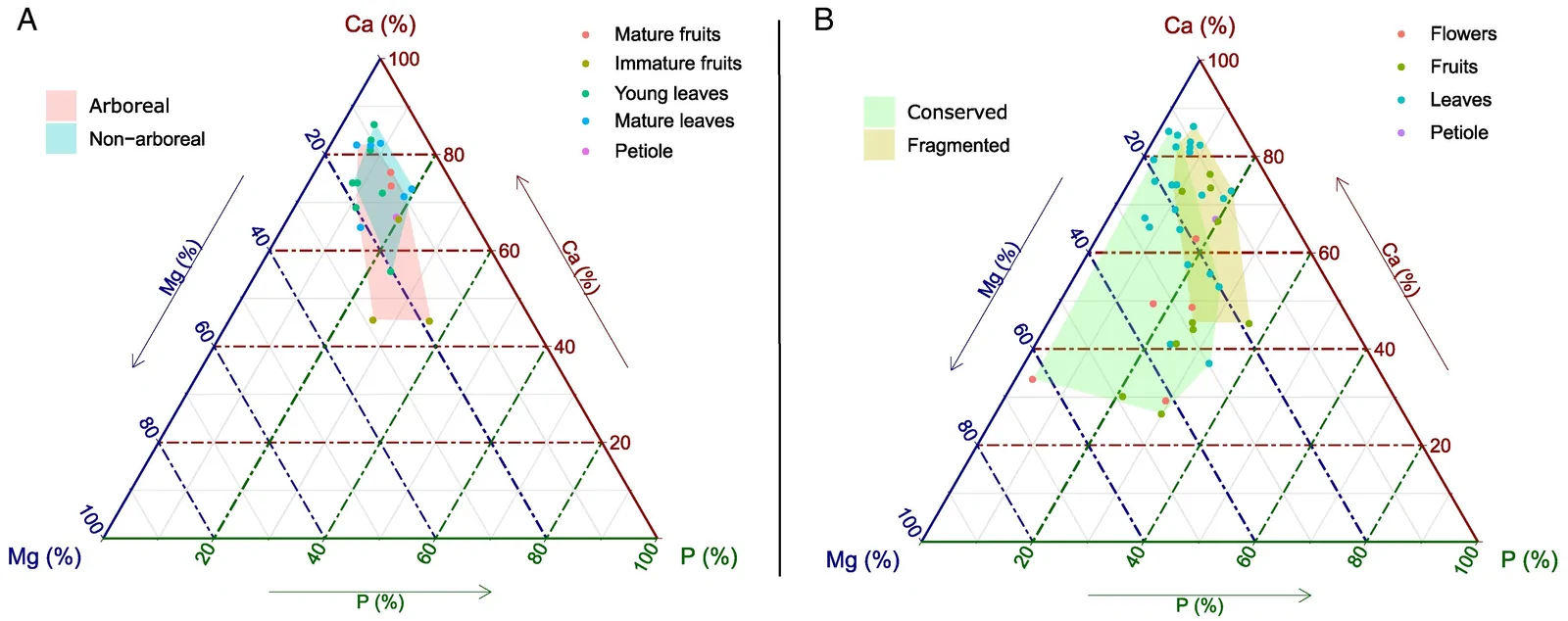
Equilateral mixture triangle showing the nutritional space (proportional contribution of the nutrient combinations available to an animal) for arboreal and non-arboreal foods (A; stage 1), and conserved and fragmented habitat foods (B; stage 1 and 3). Each point represents a species-specific food item, whose position reflects relative proportions of Ca, Mg, and P.

Foods obtained primarily from the arboreal stratum (e.g., leaves consumed in the canopy) tended to exhibit lower proportions of Ca and higher proportions of Mg and P; whereas foods obtained from the non-arboreal stratum (e.g., petioles and mature leaves consumed near the ground) tended to show higher proportions of Ca. Consistent with these patterns, absolute concentrations of Ca (*U* = 18.3; *P* < 0.05; *n* = 20; mean arboreal = 1.25%; mean non-arboreal = 2.34%), Mg (*U* = 21.1; *P* < 0.05; *n* = 20; mean arboreal = 0.23%; mean non-arboreal 0.37%), and P (*U* = 11; *P* < 0.01; *n* = 20; mean arboreal = 0.21%; mean non-arboreal = 0.34%) were significantly higher in foods obtained from the non-arboreal stratum than in those obtained from the arboreal stratum.

### Nutritional space of foods by habitat condition

On the other hand, when comparing different environments, foods from the conserved habitat occupied a broader nutritional space in terms of relative proportions of Ca, Mg, and P than foods from fragmented habitats (Fig. 2B). This larger space included foods with lower Ca proportions (<30%) and higher proportions of P (20–30%) and Mg (40–50%), indicating greater heterogeneity in mineral proportions. In contrast, foods from fragmented habitats were characterized by higher absolute concentrations of Ca and P. We found significant differences in Ca (*U* = 75.5; *P* < 0.001; *n* = 20; mean fragmented = 1.8%; mean conserved = 0.72%) and P (*U* = 58.5; *P* < 0.001; *n* = 20; mean fragmented = 0.27%; mean conserved = 0.14%).

### Mineral intake of black howler monkeys in forest fragments

The mineral intake of black howler monkeys showed significant variation both in terms of the proportion of the dry mass and in absolute terms (g/day). Ca showed the highest intake among all minerals in the diet, with an average absolute intake of 1.52%DM (Table 2), being significantly higher than P (0.6%DM) and Mg (0.08%DM). Recommended mineral intake values used for comparison were obtained from the National Research Council (NRC 2003) guidelines for captive non-human primates and are used here as a reference framework (Table 2). This pattern indicates a greater selection of Ca-rich foods, such as young leaves and ripe fruits, in terms of average g/day intake (Supplementary Material SM1). In contrast, Zn (0.21 g/day) and Cu (0.08 g/day) intake levels were lower, although within the recommended ranges for nonhuman primates (NRC, 2003).

**Table 2:** Average daily intake (% DM) of minerals in the diet of three black howler monkey groups (G-1, G-2, G-3; stage 2) inhabiting forest fragments at Balancan, Mexico, compared to recommended concentrations.

Mineral (%DM)	NRC^a^	Overall diet	Rainy season				*Nortes* season				Dry season			
			G-1	G-2	G-3	General	G-1	G-2	G-3	General	G-1	G-2	G-3	General
*g/day-food*	*—*	*181*.*0*	*163*.*5*	*131*.*3*	*192*.*6*	*163*.*7*	*118*.*7*	*176*.*8*	*138*.*9*	*149*.*13*	*201*.*6*	*190*.*04*	*318*.*05*	*240*.*56*
Cu	0.002	0.08	0.11	0.06	0.08	0.09	0.07	0.07	0.05	0.06	0.08	0.09	0.08	0.08
Mn	0.002	0.27	0.17	0.26	0.23	0.21	0.26	0.23	0.61	0.39	0.22	0.32	0.19	0.24
Fe	0.01	0.43	0.30	0.48	0.34	0.36	0.54	0.42	0.64	0.53	0.39	0.48	0.38	0.42
Zn	0.01	0.21	0.25	0.26	0.19	0.23	0.24	0.20	0.15	0.19	0.21	0.18	0.19	0.19
P	0.6	23.09	29.24	20.28	21.56	24.33	21.29	25.53	21.69	23.09	23.90	19.51	20.96	21.18
Ca	0.8	1.52	0.81	2.21	1.23	1.33	1.88	2.33	2.24	2.20	0.99	1.73	0.54	1.10
Mg	0.08	0.37	0.33	0.46	0.36	0.37	0.37	0.41	0.45	0.42	0.35	0.35	0.24	0.31

Average food intake (g/day) in italics.

^a^Recommended intakes following National Research Council (2003).

Mature and young leaves stand out as the main contributors for most minerals, including Ca, Mg, P, Fe, and Mn (Fig. 3). Young leaves are particularly relevant for P and Mg intake. Conversely, unripe and ripe fruits are more relevant for Cu intake, with unripe fruits also making a significant contribution to P and Zn intake. As shown in Table 2 and Fig. 1, black howler monkeys tended to consume more essential minerals during the *Nortes* season, particularly Ca (2.20%DM on average) and P (23.09%DM). This contrasts with the dry season, when the average Ca intake decreased to 1.10%DM. On the other hand, Mg intake remained relatively stable across all seasons (0.31–0.42%DM).

**Figure 3:**
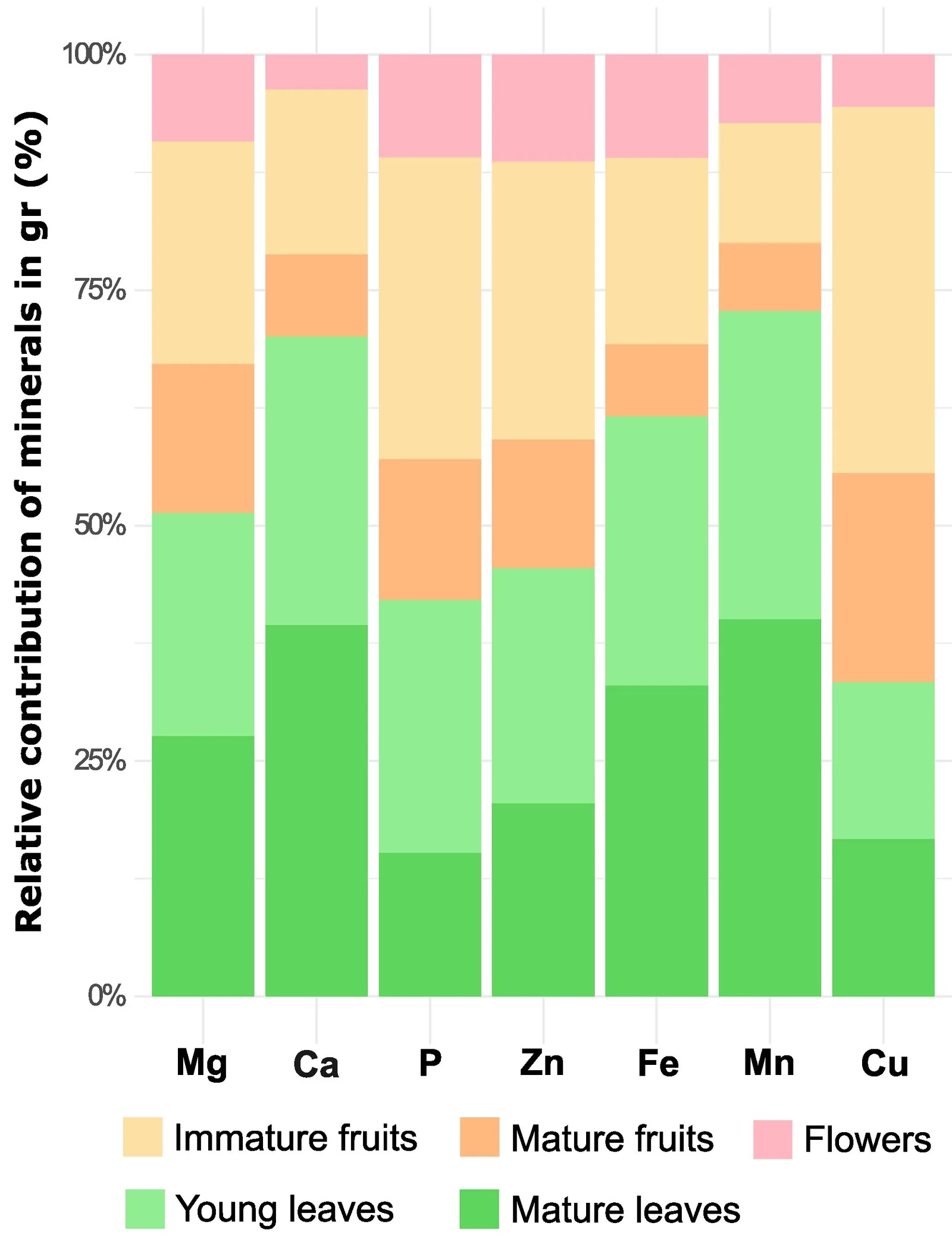
Relative mineral contribution (%) based on total grams ingested per dietary item in the diet of black howler monkeys inhabiting forest fragments at Balancan, Mexico (stage 2).

Mineral intake at Stage 2 showed distinct patterns depending on the season and sex of individuals, with significant effects of different factors (Sex, Season, and Group) found only for Ca, Mn, Fe, and Cu (Table 3). For Ca, there was a significant increase in intake during the *Nortes* season (β = 0.52, *P* < 0.01), representing a 68% increase compared to the rainy season, while the dry season (β =0.17, *P* = 0.95) showed no effect. Males had a significantly lower Ca intake, consuming 35% less (β = −0.44, *P* < 0.01) than females. For Mn, intake increased by 61% during both the *Nortes* and dry seasons (β = 0.48, *P* < 0.01), while males (β = −0.28, *P* < 0.05) showed a 24% decrease in intake. Fe intake also showed significant increases during the *Nortes* (β = 0.38, *P* < 0.01) and dry seasons (β = 0.66, *P* < 0.001), corresponding to increases of 46% and 93%, respectively. On the other hand, Cu intake decreased by 40% during the *Nortes* season (β = −0.52, *P* < 0.01), while the increase observed in the dry season (β = 0.19, *P* = 0.2) was not significant.

**Table 3:** Models using season, sex, and group to explain variation in the daily intake of minerals (g) for black howler monkey groups (G-1, G-2, G-3; stage 2) at Balancan, Mexico.

Mineral	Contrast	β-slope	SE	*t*-value	P(Chi)	Resid. dev	AIC	ΔAIC	*wi*
**Ca**									
*null model*	*—*	*—*	*—*	*—*	*—*	106.0	466.1	31.7	0
*final model*	Intercept (Rainy;Female;Group-2)	1.18	0.17	6.76	<0.001	78.6	434.4	0	1
	Season (Nortes)	0.52	0.18	2.88	<0.01				
	Season (Dry)	0.17	0.18	0.95					
	Sex (Male)	−0.44	0.15	−2.90	<0.01				
	Group-1	−0.69	0.19	−3.64	<0.001				
	Group-3	−0.58	0.18	−3.19	<0.01				
**Mn**									
*null model*	*—*	*—*	*—*	*—*	*—*	79.47	25.77	23.3	0
*final model*	Intercept (Rainy;Female;Group-2)	−1.04	0.15	−6.84	<0.001	62.25	2.46	0	1
	Season (Nortes)	0.48	0.16	3.00	<0.01				
	Season (Dry)	0.48	0.16	3.02	<0.01				
	Sex (Male)	−0.28	0.13	−2.15	<0.05				
	Group-1	−0.28	0.16	−1.68	<0.05				
	Group-3	0.20	0.16	1.31	0.1				
**Fe**									
*null model*	*—*	*—*	*—*	*—*	*—*	79.55	156.06	14.5	0
*final model*	Intercept (Rainy)	−0.65	0.10	−6.41	<0.001	66.51	142.46	0	0.6
	Season (Nortes)	0.38	0.16	2.39	<0.01				
	Season (Dry)	0.66	0.16	4.05	<0.001				
**Cu**									
*null model*	*—*	*—*	*—*	*—*	*—*	93.44	−237.1	10.2	0
*final model*	Intercept (Rainy)	−1.87	0.11	−16.85	<0.001	84.36	−247.3	0	0.8
	Season (Nortes)	−0.52	0.18	−2.96	<0.01				
	Season (Dry)	0.19	0.18	1.08	0.2				

## Discussion

In this study, we evaluated how mineral intake by black howler monkeys (*A. pigra*) varied according to food type, sex, season, canopy stratum, and habitat type. We explicitly distinguished between variation in the mineral composition of foods and variation in mineral intake derived from consumption patterns. We observed differences in food mineral composition across habitat types (fragmented vs. conserved), foraging strata (arboreal vs. non-arboreal), and seasons (rainy, dry, and *Nortes*). Leaves were the primary sources of Ca, while immature fruits showed high Mg and P content. Foods from fragmented habitats consistently exhibited higher concentrations of Ca and Mg than those from conserved habitats.

In terms of mineral intake (grams ingested per day), leaves contributed most to Ca, Fe, and Mn intake, while fruits were important sources of Cu and, to a lesser extent, P. Seasonality also had a significant impact on mineral intake, with an increase in Ca and P consumption during the *Nortes* season, and lower intake during the dry season. Ca, P, and Mg accounted for most of the variation in daily mineral intake. Together, these results underscore the importance of specific food sources and dietary flexibility, including increased leaf consumption during periods of reduced fruit availability, consistent with a complementary feeding strategy that may contribute to balancing energy and mineral intake. We further suggest that the diversity in mineral composition across food items, particularly across habitats and seasons, plays an important role in maintaining nutritional balance in black howler monkeys and highlights the relevance of conserving habitat heterogeneity to support their long-term health and persistence.

Among all the food sources exploited by howler monkeys in our study, lianas were more prevalent in fragmented habitats, serving as key food resources, as previously reported for primates in disturbed forests (Dunn et al. 2012). In our study, young leaves and lianas were highly consumed by howlers and exhibited the highest concentrations of Ca, a pattern consistent with findings in other highly folivorous primates such as diademed sifakas (*P. diadema*), which also rely heavily on young and mature leaves (Irwin et al. 2017). In contrast, other folivorous primates, such as *C. angolensis palliatus*, consume leaves with higher P and lower Ca content (Dunham and Opere 2021), indicating that mineral profiles of consumed foods vary across species and ecological contexts.

In addition, immature fruits and lianas contributed to Cu and P intake, suggesting that different food types complement one another in shaping overall mineral intake. We also observed that groups in the fragmented habitats consumed foods with higher concentrations of Fe, P, Mn, and K during the N*ortes* season, reflecting seasonal shifts in diet composition rather than fixed dietary preferences. These patterns highlight the importance of continuous monitoring of these changes to better understand the dietary strategies of primates.

### Habitat type and mineral heterogeneity

Food mineral concentrations in absolute terms were higher in the fragmented habitat compared to the conserved habitat. This is consistent with findings in sifaka groups from disturbed sites, where some minerals were found in higher concentrations; however, this did not necessarily result in greater mineral intake due to a lower overall food consumption (Irwin et al. 2017). Similarly, guerezas (*C. guereza*) in Kakamega Forest, Kenya, exploit Na-rich trees and soil patches only found near disturbed areas, suggesting that edge resources offer enhanced mineral profiles compared to conserved habitats (Fashing et al. 2007). While direct comparisons are not possible due to differences in species and ecological context, these findings are consistent with the pattern observed in our study, in which conserved habitats provided a more diverse mineral landscape than fragmented forests.

These patterns highlight how fragmented environments commonly provide access to food resources from both native and introduced plant species, which may differ in mineral composition depending on species identity and local soil conditions (Chapin 1980; Chaves and Bicca-Marques 2012; Pozo-Montuy et al. 2013), allowing howler monkey populations to access a distinct nutritional profile, with higher concentrations of certain minerals despite lower dietary diversity. In contrast, conserved habitats provided foods that, although lower in absolute terms, offered greater taxonomic and functional diversity, resulting in a broader nutrient spectrum.

Comparable dietary and behavioral adjustments in response to habitat fragmentation or degradation have been documented in other primates. For example, orangutans (*Pongo abelii*) inhabiting human-modified and degraded landscapes can increase their reliance on non-typical or lower-preference resources, including crop foods and bark, illustrating behavioral flexibility under altered resource regimes (Campbell-Smith et al. 2011). This shift demonstrates a critical behavioral adaptation to reduced resource availability. Similarly, Taihangshan macaques (*Macaca mulatta tcheliensis*) living in highly seasonal northern montane habitats were found to employ a seed-based feeding strategy, with tree leaves and herbaceous leaves serving as fallback foods when their preferred seed resources were scarce. This reflects their ecological adaptation to low-quality food environments characterized by strong seasonal changes (Shao et al. 2023).

### Foraging strata and access to mineral-rich foods

Terrestriality in arboreal primates can be influenced by multiple ecological and behavioral factors (Eppley et al. 2022), among which non-arboreal foraging plays a central role. Many primates occasionally descend to the ground to access water sources or mineral-rich substrates, including through geophagy, defined as the deliberate ingestion of soil or sediment to supplement mineral intake or buffer dietary imbalances (Link et al. 2011). These behaviors indicate that terrestrial resources can complement arboreal diets, particularly in environments where resource availability is spatially heterogeneous.

Previous studies have shown that folivorous and generalist primates may be better adapted to take advantage of resources from the terrestrial stratum (Eppley et al. 2016). For example, Japanese macaques and mountain gorillas obtain substantial amounts of minerals from herbaceous leaves, with mineral concentrations varying according to food type and foraging context (Cancelliere et al. 2014; Hanya et al. 2023). In our study, we found that while in a conserved habitat the nutritional niche is broader (Fig. 2), black howler monkeys in fragmented habitats obtained high concentrations of Ca from non-arboreal foods, including leaves and petioles from lianas. This pattern suggests that occasional use of lower strata may facilitate access to mineral-rich food items in structurally simplified or fragmented landscapes. Descending to the ground may represent a flexible response to seasonal food (e.g., fruit) scarcity, enabling primates to expand their trophic niche and reduce competition for arboreal resources in less diverse ecosystems. Overall, these results underscore the role of terrestrial foraging as a component of nutritional niche flexibility in fragmented landscapes.

### Seasonality and mineral intake patterns

In this study, the *Nortes* season was associated with the highest absolute intake of essential minerals such as Ca and P, while the dry season recorded lower average intake levels. This seasonal increase in mineral intake may reflect shifts in food availability rather than active regulation, as the prevalence of young leaves and fallback foods such as petioles, both more frequently consumed during the *Nortes* season and relatively rich in P, may incidentally increase mineral intake (Aristizabal et al. 2019). Similar seasonal patterns have been observed in other folivorous primates. For instance, diademed sifakas (*P. diadema*) increase Ca and P intake during periods of higher food availability (Irwin et al. 2017), paralleling the pattern observed in black howler monkeys. In contrast, Angolan black-and-white colobus monkeys (*C. angolensis palliatus*) selectively consume leaves with higher P and lower Ca concentrations (Dunham and Opere 2021), suggesting different mineral prioritization or availability in their habitat. Sodium intake remained consistently low across seasons in our study, a pattern also reported for colobines in Kibale National Park that consume Na-poor foods and show limited compensatory mechanisms (Rode et al. 2003). However, when alternative Na-rich resources are accessible, such as bark from *Eucalyptus* trees planted near forest edges, red colobus monkeys (*Piliocolobus tephrosceles)* have been observed to exploit these fallback foods frequently, despite their high content of secondary metabolites (Lauer et al. 2025). These behavioral strategies illustrate the importance of complementary nutrients beyond protein and fiber in folivores and further suggest that unusual or non-native plant parts may play key roles in mineral acquisition under limiting ecological conditions. While Japanese macaques (*Macaca fuscata*) are more generalist, they also display selective foraging for mineral-rich terrestrial herbs and have shown the ability to seek out higher-Na or Ca foods when needed (Hanya et al. 2023). Unlike macaques, however, black howler monkeys in our study appear to meet mineral requirements, especially for Ca, through non-arboreal liana leaves rather than herbaceous plants. Together, these comparisons highlight shared seasonal adjustments among folivores, while also underscoring species-specific and habitat-driven differences in how mineral constraints are addressed.

### Mineral balance and reference values in wild primates

Mineral deficiencies are difficult to detect in wild primates, as comparisons with dietary recommendations established for captive individuals, such as those proposed by the National Research Council (NRC 2003), often suggest insufficient mineral acquisition (Irwin et al. 2017). In our study, Na concentrations in foods consumed by howlers were consistently low in both forest fragments (0.01%DM) and conserved habitat (0.04%DM), falling well below NRC recommendations for non-human primate: 0.25–0.65%DM. Sodium plays a central role in osmotic regulation and nerve transmission; however, many plant foods consumed by wild primates are naturally Na-poor (Rothman et al. 2006; Shimwa et al. 2024). Similar patterns have been documented in other primate populations. For instance, colobine monkeys in Kibale National Park consume extremely low diets in Na and appear to show limited behavioral or physiological compensation, likely reflecting low environmental availability of this mineral (Rode et al. 2003). Na limitation has been proposed as an important ecological constraint, with broader implications for herbivore abundance and population dynamics in terrestrial ecosystems (Welti et al. 2019). In this context, Na scarcity may influence feeding behavior and access to critical resources, ultimately affecting population persistence (Hanya and Chapman 2013).

Evidence from wild primate populations further suggests that mineral availability can shape dietary resilience under environmental disturbance. For example, in black howler monkeys in Belize, the loss of fig trees following a hurricane reduced access to important sources of Ca and Na, leading to significant dietary changes and long-term population consequences (Behie and Pavelka 2012). In sum, mineral balance in wild primates must be interpreted within an ecological framework that considers environmental availability and dietary context, rather than strict adherence to captive reference values.

### Physiological considerations of mineral excess and imbalance

In our study, Ca, P, and Mg accounted for most of the variation in daily mineral intake and were ingested at levels that, in some cases, exceeded dietary recommendations established for captive primates by the NRC (2003). However, comparisons with captive reference values should be interpreted with caution, as mineral concentrations in wild foods are often below NRC standards without apparent negative effects on wild primate populations (Irwin et al. 2017; Dunham and Rodriguez-Saona 2018). Rather than indicating mineral imbalance, these patterns suggest that primates may rely on physiological mechanisms that allow them to maintain nutritional homeostasis (e.g., endocrine regulation of Ca-P balance, modulation of intestinal absorption efficiency, renal excretion of excess minerals) when consuming diets with variable mineral composition (Barboza et al. 2009; Hanya et al. 2023). Such flexibility may help buffer both deficiencies and surpluses, particularly in seasonal or heterogeneous environments. In this context, the relatively high Ca intake observed in our study does not necessarily imply adverse effects, although excessive Ca intake has been associated in other animal models with altered P metabolism and increased renal load (Goto and Sawamura 1973  Lipkin 1998; Brugger and Liesegang 2024). Seasonal increases in P intake, such as those observed during the *Nortes* season, may further influence Ca metabolism through endocrine pathways involving parathyroid hormone (Chang and Anderson 2017; Lamberg-Allardt and Kemi 2017), but the relevance of these mechanisms in wild primates remains difficult to assess due to the lack of direct physiological measurements.

Importantly, mineral intake alone does not fully capture mineral balance, as bioavailability can be strongly influenced by anti-nutritional compounds such as tannins, which may reduce mineral absorption and effective intake, as documented in other primate species (Cancelliere et al. 2014). Information on bioavailability in nonhuman primates is limited (Schmidt et al. 2010; Hanya et al. 2023), and digestibility data would be necessary to estimate mineral balance. However, noninvasive approaches such as fecal mineral analyses, which allow estimation of apparent absorption through intake-excretion comparisons, the analysis of minerals in keratinized tissues (e.g., hair) (Kok et al. 1990) which can reflect longer-term mineral incorporation, and the analysis of electrolyte excretion in urine, may provide valuable proxies for assessing mineral bioavailability in wild primates without the need for capture or experimental manipulation (Cancelliere et al. 2014; Jutha et al. 2022).

In primates such as other atelids and chimpanzees, behavioral strategies such as geophagy or the use of mineral-rich substrates (e.g., clay or termite mounds) have been reported as responses to mineral deficiency, particularly for Na (Hladik and Gueguen 1974; Link et al. 2011; Reynolds et al. 2019). Although we did not document such behaviors in our study, these examples illustrate the diversity of physiological and behavioral pathways through which primates may cope with mineral constraints. Taken together, our results indicate that mineral consumption contributes to nutritional balance as an integrated consequence of dietary composition and foraging decisions, rather than as an independent determinant of food choice.

### Soil–plant pathways and mineral availability

Mineral concentrations in plant foods are strongly influenced by edaphic factors, including soil mineral content, pH, and organic matter (Harper and Belnap 2001; Lee and Lee 2014). Several studies have shown that the uptake of elements such as Ca, Mg, Fe, and Mn by plants is closely linked to their availability in the soil, with direct correlations reported between soil and foliar mineral concentrations (Roca-Pérez et al. 2013; Irwin et al. 2017). Soil pH further modulates nutrient availability, with Ca and Cu better absorbed in alkaline soils, and Mn and Zn preferentially absorbed in acidic soils (Bravo et al. 2017).

In the fragmented habitat of Balancán, Tabasco, the predominant soil types include neutral and flat soils prone to flooding (Muñoz et al. 2007; Castelán-Estrada et al. 2013). Therefore, plants in this area are likely to absorb more Ca, leading folivorous primates living there to consume large amounts of this mineral. In contrast, the conserved habitat of El Tormento, located on the karstic limestone platform of the Yucatán Peninsula, is characterized by shallow soils, high pH, and low organic matter, conditions that can limit the availability of P and micronutrients such as Fe and Zn due to leaching and carbonate binding (Perry et al. 2003; Leytem and Mikkelsen 2005; Taalab et al. 2019). These soil characteristics were likely responsible for the presence of condensed tannins, low protein-to-fiber ratio (<0.45), low Fe content, and moderate P concentration in the foliage at El Tormento (Righini et al. 2017).

### Conservation and management implications

Although fragmented habitats provided foods with higher concentrations of certain minerals such as Ca and Fe, Na availability remained low, underscoring the importance of maintaining plant diversity and habitat heterogeneity to support balanced mineral intake. Additionally, our observations also highlight the limitations of applying dietary recommendations developed for captive primates to wild populations, emphasizing the need for context-specific nutritional frameworks that account for environmental variability. Integrating information on mineral intake, bioavailability, and habitat characteristics into conservation planning may improve management strategies for black howler monkeys and other primates living in fragmented and conserved landscapes. A better understanding of mineral requirements and dietary flexibility in wild primates can also inform captive diet formulation and contribute to long-term species conservation under ongoing environmental change (Irwin et al. 2017; Hanya et al. 2023).

Importantly, mineral intake should be interpreted as part of an integrated nutritional strategy rather than as an isolated driver of food selection. In primates, and particularly in howler monkeys, dietary decisions reflect the simultaneous balancing of minerals with other nutritional dimensions, including macronutrients, fiber content, secondary compounds, and food availability (Righini et al. 2017). Thus, the contribution of minerals to nutritional balance emerges from the interaction between dietary composition and foraging decisions as a whole, rather than from mineral intake alone.

## Supplementary Material

zoag004_Supplementary_Data
