# Supplementary material for: Mineral sources in natural habitats for a leaf-eating primate
Source: Curr Zool. 2026 Jan 29;72(5):678–91. doi: 10.1093/cz/zoag004 (PMC13690592; doi:10.1093/cz/zoag004)
Supplement: Supplementary file 1 [file zoag004_supplementary_data.docx]

**FIGURE SM1.** Average intake of absolute content of minerals (g of dry matter) per feeding event in 44 black howler monkey foods at Balancán, Mexico (Stage 2). Boxes represent the average intake, and the error bars the standard deviation.
